# Uncertainty‐suppressed long‐tailed learning with lightweight feature adaptation for multi‐label chest X‐ray classification

**DOI:** 10.1002/acm2.70873

**Published:** 2026-10-20

**Authors:** Lu Li

**Affiliations:** ^1^ Department of Radiology Hubei Province Academy of Traditional Chinese Medicine, Hubei Provincial Hospital of Traditional Chinese Medicine (Guanggu Campus) Wuhan Hubei Province China

**Keywords:** chest radiograph classification, feature adaptation, long‐tailed learning, medical image analysis, multi‐label classification, uncertain labels

## Abstract

**Background:**

Report‐mined chest radiograph datasets commonly contain both long‐tailed label distributions and uncertain annotations. Conventional frequency‐based re‐weighting can strengthen rare‐label learning, but it may also amplify unreliable supervision when uncertain labels occur in minority classes.

**Purpose:**

To develop and evaluate an uncertainty‐suppressed long‐tailed learning method with lightweight feature adaptation for multi‐label chest X‐ray (CXR) classification under imbalanced and uncertain report‐derived supervision.

**Methods:**

The proposed uncertainty‐suppressed long‐tailed feature adaptation (USLFA) method combines a lightweight label‐adaptive feature head with an uncertainty‐suppressed long‐tailed loss. The primary analysis used a patient‐level 70%/10%/20% split of CheXpert for 14 observation labels. Robustness was further examined across two additional patient‐level partitions, on the recommended MIMIC‐CXR‐JPG test split without retraining, and on the official radiologist‐annotated CheXpert test set. Performance was assessed using macro‐AUROC, macro‐AUPRC, macro F1, frequency‐group analysis, ablation studies, and uncertainty‐handling comparisons. Ninety‐five percent confidence intervals (CIs) were estimated by patient‐level bootstrap resampling, and formal pairwise tests in the primary CheXpert analysis used paired permutation testing with Holm correction.

**Results:**

On the primary CheXpert split, USLFA achieved a macro‐AUROC of 0.864 (95% CI, 0.856–0.871), macro‐AUPRC of 0.547 (95% CI, 0.532–0.560), and macro F1 of 0.501 (95% CI, 0.487–0.514). Compared with ConvNeXt‐Tiny using binary cross‐entropy (BCE), the gains in macro‐AUPRC and macro F1 were both 0.051 and remained significant after Holm correction (both *p <* 0.001). Compared with label‐distribution‐aware margin (LDAM), USLFA improved macro‐AUPRC by 0.018 (Holm‐adjusted *p =* 0.024) and macro F1 by 0.017 (Holm‐adjusted *p =* 0.041). The ordering was preserved across three patient‐level partitions, with USLFA macro‐AUPRC ranging from 0.540 to 0.555. On MIMIC‐CXR‐JPG, USLFA achieved a macro‐AUPRC of 0.485 (95% CI, 0.467–0.502), and on the official CheXpert test set it achieved a mean AUROC of 0.920 (95% CI, 0.908–0.931) across the five competition observations.

**Conclusions:**

USLFA improved macro‐AUPRC and rare‐label recognition without introducing an additional inference‐time branch. Macro F1 was significantly higher than the principal BCE and LDAM baselines in the main comparison, whereas the additional macro F1 gain attributable to uncertainty suppression over U Soft was not significant after Holm correction. The repeated‐split, external‐dataset, and official‐test analyses provide complementary evidence that the observed advantage is not confined to a single custom partition.

## 1 INTRODUCTION

Chest radiography remains one of the most frequently used imaging examinations in clinical practice, and automatic chest X‐ray (CXR) recognition has progressed from single disease screening toward multi‐label abnormality detection. Recent reviews show that the field is moving from conventional convolutional pipelines to transformer, foundation, and multimodal systems, but they also emphasize persistent concerns about generalization, label quality, and explainability.^1^ These concerns are particularly visible in long‐tailed CXR benchmarks, where a few common findings dominate the training set while many clinically meaningful observations appear rarely.^2^ The recent Chest X‐ray Long‐Tailed (CXR‐LT) 2026 challenge further extends this setting to multi‐center and zero‐shot evaluation, indicating that rare finding recognition remains an open problem rather than a solved benchmark detail.^3^ Large public datasets have made CXR classification reproducible, but their annotation mechanisms also define the limitations of the task. CheXpert provides 224,316 chest radiographs with uncertainty labels extracted from reports, making it a representative dataset for studying report‐mined supervision.^4^ MIMIC‐CXR‐JPG provides 377,110 images with structured labels derived from free‐text radiology reports, which further shows the scale at which report‐derived labels are now used.^5^ ChestX‐ray14 and PadChest enlarge the available disease space and institutional diversity, but both also rely on weak or partially automated labeling pipelines,^6^, ^7^ Therefore, the problem addressed in this work is not only how to train a stronger classifier, but also how to train under label imbalance and annotation uncertainty at the same time.

Recent model development has improved CXR representation learning. EVA‐X and CheXFound show that self‐supervised or foundation‐style pretraining can provide transferable CXR features, and BiomedCLIP demonstrates the value of large biomedical image‐text pretraining,^8^, ^9^, ^10^ At the task‐specific level, graph‐guided cross attention, attribute‐aware semantic graphs, and long‐range dependency modeling have been used to capture lesion heterogeneity and label correlations in multi‐label classification,^11^, ^12^, ^13^ Multi‐task and relation‐aware designs have also been proposed to combine disease recognition with localization or dynamic label reasoning,^14^, ^15^, ^16^ In parallel, label relation modeling has been widely explored because thoracic findings are not independent. Graph convolutional methods explicitly propagate disease co‐occurrence information, while global‐local correlation modeling attempts to describe both dataset‐level and sample‐level dependencies,^17^, ^18^ Recent weak‐label settings further show that CXR classifiers may have to learn from incomplete labels or projection‐specific distributions rather than fully curated annotations,^19^, ^20^ These advances support the importance of feature adaptation and label‐specific modeling, but many of them still optimize all labels under the assumption that the available positive and negative annotations are equally reliable.

Long‐tailed learning methods commonly increase the training contribution of minority classes. Class‐balanced loss (CBL) uses the effective number of samples to reweight categories, and label‐distribution‐aware margin (LDAM) introduces LDAMs for imbalanced recognition,^21^, ^22^ Asymmetric loss (ASL) and focal loss address different forms of positive‐negative or easy‐hard imbalance, while distribution‐balanced loss is specifically designed for multi‐label datasets with label co‐occurrence,^23^, ^24^, ^25^ These methods are useful baselines, but frequency‐driven weighting can be risky in report‐mined CXR classification. If a tail‐class annotation is uncertain, increasing its weight without considering its reliability may amplify noisy gradients rather than improve rare‐label learning. Several recent works have begun to address label reliability and benchmark limitations. A label‐wise reliability‐aware classifier was proposed for robust CXR multi‐label learning, and a recent analysis of public CXR datasets highlighted label quality, domain shift, bias, and evaluation weaknesses,^26^, ^27^ CheXbert improves report label extraction by combining automatic labelers and expert annotations, while CheXpert Plus and PadChest‐GR expand the available paired image‐text or grounded information for radiology research,^28^, ^29^, ^30^ Independent studies on domain shift and external validation also show that good internal performance may not translate directly across populations, sites, or clinical workflows,^31^, ^32^, ^33^ These observations motivate a more cautious use of long‐tailed reweighting when the label state is uncertain.

This paper proposes Uncertainty‐Suppressed Long‐Tailed Feature Adaptation, abbreviated as USLFA, for multi‐label CXR classification. The core idea is to enhance rare‐class supervision while reducing the influence of uncertain annotations. To implement this idea without introducing a heavy architecture, USLFA uses a lightweight label‐adaptive feature head and an uncertainty‐suppressed long‐tailed loss. The proposed method is positioned between ordinary class‐balanced learning and label reliability modeling. It does not attempt to build a new foundation model or a separate reliability network. Instead, it focuses on the interaction between class frequency and annotation uncertainty in report‐mined CXR datasets. The main contributions of this work are threefold. First, we identify the risk that frequency‐only long‐tailed reweighting may amplify uncertain supervision in report‐mined CXR datasets. Second, we propose an uncertainty‐suppressed long‐tailed learning strategy that combines class‐frequency priors with label uncertainty states, so that rare labels are strengthened only when the supervision is sufficiently reliable. Third, we design a lightweight label‐adaptive feature head that improves label‐specific discrimination without relying on a heavy multi‐branch network, and we evaluate the method through overall and frequency‐group comparisons, component and uncertainty‐handling analyses, repeated patient‐level partitions, and independent evaluations on MIMIC‐CXR‐JPG and the official CheXpert test set.

## 2 RELATED WORK

### 2.1 Multi‐label CXR classification

Early deep learning systems for CXR classification established the feasibility of image‐level abnormality detection, but also exposed issues of dataset shift and label noise. Cross‐institution pneumonia detection demonstrated that a model trained in one environment may lose performance when evaluated elsewhere.^34^ CheXNet and CheXNeXt then showed that DenseNet‐style architectures could reach strong disease recognition performance on large CXR datasets and radiologist comparison settings,^35^, ^36^ Later approaches moved beyond a single shared classifier by introducing category‐wise residual attention,^37^ label co‐occurrence graph modeling,^17^ and global‐local label correlation modeling.^18^ Recent CXR classification work is increasingly focused on visual heterogeneity and label‐specific reasoning. Graph‐guided multiscale cross attention integrates heterogeneous visual representations with label reasoning, while the multi‐scale and attribute‐aware semantic graph (MSASG) method combines multi‐scale feature reconstruction with attribute‐aware semantic graph modeling,^11^, ^12^ Long‐range dependency capture and adaptive label‐relation graph methods further indicate that global context and dynamic disease relationships are useful for multi‐label recognition,^13^, ^15^ In parallel, multi‐task frameworks such as CXR‐MultiTaskNet use localization and Grad‐CAM‐based explainability to improve the interpretability of disease recognition.^14^ These methods strengthen visual and semantic representation, but their training objectives usually treat label imbalance and uncertainty as separate issues.

Another line of work uses weak supervision, vision‐language alignment, or class‐specific tokens to reduce dependence on dense annotations. Single‐positive multi‐label learning studies incomplete annotation settings in CXR recognition, while GLoRIA, CheXzero, and MedCLIP use image‐text alignment or self‐supervised pretraining to improve label‐efficient and zero‐shot recognition,^19^, ^38^, ^39^ MedCLIP further shows that unpaired medical images and text can be used for scalable contrastive learning.^40^ CLARiTy uses class‐specific tokens and attention refinement for multi‐label classification and weak localization, and attention‐based X‐ray models with knowledge distillation have also been proposed to improve multi‐label diagnosis with compact inference,^41^, ^42^ These methods are related to the label‐adaptive head in USLFA because they emphasize label‐specific evidence. The difference is that USLFA keeps the head lightweight and focuses the main contribution on uncertainty‐suppressed long‐tailed optimization rather than introducing another large transformer or localization module.

### 2.2 Long‐tailed learning and uncertain labels

Long‐tailed recognition has been studied extensively in natural image classification and multi‐label recognition. The CBL reweights classes according to the effective number of samples, and LDAM adjusts class margins according to label frequency,^21^, ^22^ Focal loss reduces the effect of easy samples, while ASL improves multi‐label optimization by treating positive and negative gradients differently,^23^, ^24^ Distribution‐balanced loss considers label co‐occurrence and negative dominance in long‐tailed multi‐label datasets.^25^ These methods provide important baselines for CXR classification, but their weighting mechanisms are usually driven by class statistics or prediction difficulty, not by whether the label itself is uncertain. This distinction is important in CXR datasets because many labels are extracted from reports instead of being manually verified for every image. CheXpert explicitly represents uncertain observations, MIMIC‐CXR‐JPG provides structured labels derived from free‐text reports, and CheXbert improves automatic report labeling using BERT and expert annotation signals,^4^, ^5^, ^28^ Recent reliability‐aware CXR classification directly models label‐wise reliability, showing that incomplete and noisy report‐derived labels can affect robust multi‐label training.^26^

The interaction between label imbalance and uncertainty is also reflected in recent CXR benchmarks. CXR‐LT 2024 includes a large noisy test set, a manually annotated gold standard subset, and a zero‐shot task, while CXR‐LT 2026 extends the setting to multi‐center data and unseen rare findings,^2^, ^3^ Projection‐aware CXR‐LT solutions also combine multi‐label classification with zero‐shot strategies, indicating that rare‐label recognition is increasingly evaluated under realistic distribution shifts.^20^ In this context, a method that only increases tail‐class gradients may improve some rare labels but can remain unstable when the rare label has a high uncertain‐label ratio. USLFA differs from a reliability classifier in its scope. It does not infer a new label state through an additional branch. Instead, it uses the observed uncertainty state to suppress the effective training weight when class‐frequency reweighting would otherwise overemphasize an uncertain rare label.

### 2.3 Foundation models, vision‐language learning, and generalization

Foundation and vision‐language models have changed the way CXR representations are learned. EVA‐X and CheXFound demonstrate that large‐scale self‐supervised CXR pretraining can provide strong transferable image features for multiple downstream tasks,^8^, ^9^ CheXzero^39^ and MedCLIP^40^ show that image‐report or medical image‐text contrastive learning can improve label‐efficient CXR recognition, while BiomedCLIP^10^ further scales biomedical vision‐language pretraining to broader image‐text pairs. These models suggest that the feature extractor can be strengthened substantially, but even strong features still require task‐level adaptation when the downstream labels are long‐tailed and uncertain. Recent dataset extensions and grounded radiology resources also point toward more reliable evaluation. CheXpert Plus augments CheXpert with reports, demographics, image formats, and additional metadata, while PadChest‐GR provides bilingual grounded annotations for image‐report research,^29^, ^30^ Domain shift analyses in CXR classification show that label quality, demographic factors, and acquisition conditions can affect performance across populations and institutions,^31^, ^32^ External validation studies therefore remain necessary even when internal discrimination is strong.^33^ These observations are distinct from the label‐reliability problem addressed by USLFA: the proposed loss controls the influence of uncertain report‐derived supervision during training, whereas cross‐dataset generalization must be evaluated empirically.

Explainability and bias analyses are relevant because a long‐tailed model may improve numerical metrics while still relying on unstable image regions. Recent prompt‐based zero‐shot classification studies of CXR reports further show that model performance can depend on prompt design and report content.^43^ Grad‐CAM remains a common tool for qualitative inspection of class activation maps, but it should be interpreted as visual evidence rather than lesion‐level ground truth.^44^ Bias analyses of chest radiography foundation models show that large‐scale pretraining does not automatically remove dataset‐related risk.^45^ Therefore, this study combines quantitative evaluation with group‐wise analysis, uncertain‐label strategy comparison, and qualitative Grad‐CAM visualization. The Grad‐CAM analysis is used only as supplementary evidence for whether the proposed training strategy encourages more stable disease‐related attention patterns.

## 3 METHOD

The proposed method is named as USLFA. It is developed for multi‐label CXR classification under long‐tailed disease distributions and uncertain report‐mined annotations. The design follows two considerations. First, a classifier should learn label‐specific evidence without relying on a heavy multi‐branch network. Second, rare‐class re‐weighting should not be applied blindly when the corresponding annotation is uncertain. USLFA therefore contains a lightweight label‐adaptive feature head and an uncertainty‐suppressed long‐tailed loss. Figure 1 shows the overall framework.

**FIGURE 1:**
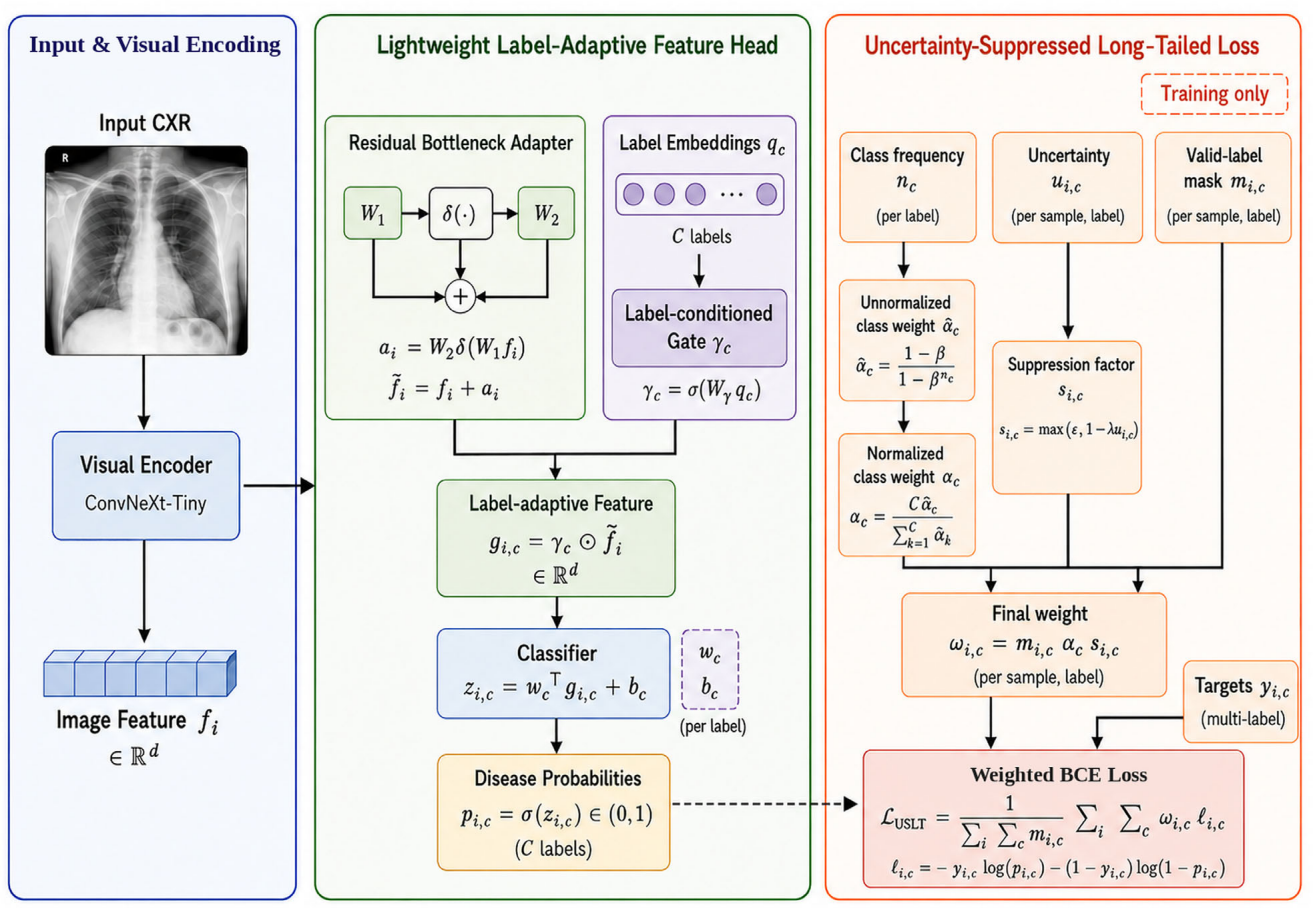
Overall framework of USLFA, including the visual encoder, the lightweight label‐adaptive feature head, the multi‐label prediction layer, and the uncertainty‐suppressed long‐tailed training loss.

### 3.1 Overall framework and notation

For CheXpert annotations, let $r_{i,c}\in \left\{1,0,-1,\emptyset \right\}$ denote the raw state of the c‐th label for the i‐th image, where 1, 0, ‐1, and ∅ indicate positive, negative, uncertain, and blank states, respectively. In USLFA, the raw label state is converted into the training target $y_{i,c}$, the uncertainty indicator $u_{i,c}$, and the valid‐label mask $m_{i,c}$, then $r_{i,c}=1:\;\left(y_{i,c},u_{i,c},m_{i,c}\right)=\left(1,0,1\right)$, $r_{i,c}=0\;\text{|or|}\;r_{i,c}=\emptyset :\left(y_{i,c},u_{i,c},m_{i,c}\right)=\left(0,0,1\right)$ and $r_{i,c}=-1:\;\left(y_{i,c},u_{i,c},m_{i,c}\right)=\left(0.5,1,1\right)$. In the main protocol, blank states are treated as negative observations following a commonly used CheXpert label‐conversion setting,^4^ while the valid‐label mask is retained for strategies that ignore uncertain or invalid sample‐label pairs. Let the training set be written as:

(1) $$\begin{array}{cc} D & ={\left\{\left(x_{i},y_{i},u_{i},m_{i}\right)\right\}}_{i=1}^{N},y_{i}\in {\left\{0,1\right\}}^{C},u_{i}\in {\left\{0,1\right\}}^{C},\\ & m_{i}\in {\left\{0,1\right\}}^{C}. \end{array}$$

where, D denotes the training set, N is the number of CXR images, and C is the number of observation labels. The vector $x_{i}$ is the i‐th image, $y_{i}$ is the multi‐label target, $u_{i}$ records the annotation uncertainty of each label, and $m_{i}$ is a valid‐label mask. When $m_{i,c}=0$, the c‐th label of the i‐th image is excluded from the loss.

For each image, a visual encoder extracts a compact feature representation,

(2) $$\;f_{i}=E_{\theta }\;\left(x_{i}\right),\;f_{i}\in R^{d}.$$

where, $E_{\theta }\left(\cdot \right)$ denotes the image encoder with parameters θ, $f_{i}$ is the extracted feature, and d is the feature dimension. The encoder can be a conventional convolutional backbone or a model initialized from CXR pretraining. The proposed head and loss do not depend on a specific backbone.

### 3.2 Lightweight label‐adaptive feature head

A standard multi‐label classifier maps one shared image feature to all label probabilities. This form is efficient, but it assumes that the same global feature is equally suitable for all labels. In CXR classification, different observation labels often rely on different visual cues. Cardiomegaly is related to the cardiac silhouette, while pleural effusion and atelectasis are more dependent on different local patterns. To introduce label‐specific adaptation with limited additional parameters, USLFA first uses a residual bottleneck transformation,

(3) $$\;a_{i}=W_{2}\;\delta \left(W_{1}f_{i}\right),\;\;{\tilde{f}}_{i}=f_{i}+a_{i}.$$

where, $W_{1}\in R^{h\times d}$ and $W_{2}\in R^{d\times h}$ are learnable projection matrices, δ(·) is a nonlinear activation function, $a_{i}$ is the residual adaptation term, and ${\tilde{f}}_{i}$ is the adapted image feature. The residual form preserves the original visual representation while allowing task‐specific adjustment.

Each observation label is associated with a learnable embedding. The embedding generates a label‐conditioned channel gate,

(4) $$\gamma_{c}=\sigma \left(W_{\gamma }q_{c}\right),\;c=1,2,\cdots ,C.$$

where, $q_{c}\in R^{e}$ is the embedding of the c‐th observation label, $W_{\gamma }$ is a learnable projection matrix, σ(·) is the sigmoid function, and $\gamma_{c}$ is the label‐conditioned gate. This gate allows different observation labels to emphasize different feature dimensions.

The feature used for the c‐th label and its logit are calculated by

(5) $$g_{i,c}=\gamma_{c}⊙{\tilde{f}}_{i},\;z_{i,c}=w_{c}^{⊤}g_{i,c}+b_{c}.$$

where, ⊙ denotes element‐wise multiplication, $g_{i,c}$ is the label‐adapted feature, $w_{c}$ and $b_{c}$ are the classifier weight and bias for the c‐th label, and $z_{i,c}$ is the corresponding logit. The prediction probability is

(6) $$\;p_{i,c}=\sigma \;\left(z_{i,c}\right)=\frac{1}{1+\exp\left(-z_{i,c}\right)}\;.$$

where, $p_{i,c}$ denotes the predicted probability of the c‐th observation label for the i‐th image. The same output is used during training and testing, so the proposed head introduces no extra inference branch.

### 3.3 Uncertainty‐suppressed long‐tailed loss

Long‐tailed learning usually increases the contribution of rare labels according to their frequencies. This strategy can improve rare‐label recognition when labels are reliable. In report‐mined CXR datasets, however, uncertain and incomplete annotations are common. If a tail class has many uncertain labels, directly applying a large class weight may also strengthen unreliable supervision. USLFA addresses this issue by combining the class‐frequency prior with an uncertainty suppression factor.

For each valid sample‐label pair, the basic binary loss is

(7) $$\;\ell_{i,c}=-y_{i,c}\log\left(p_{i,c}\right)-\left(1-y_{i,c}\right)\log\left(1-p_{i,c}\right).$$

where, $\ell_{i,c}$ is the binary cross‐entropy (BCE) loss for the c‐th label of the i‐th image, $y_{i,c}$ is the training target after label‐state conversion, and $p_{i,c}$ is the predicted probability. Since $y_{i,c}\in \left[0,1\right]$, this formulation supports both hard labels and soft targets for uncertain labels. The positive sample count of each label is computed as

(8) $$\;n_{c}=\sum_{i=1}^{N}m_{i,c}\;I\left(\;r_{i,c}=1\right).$$

where, $n_{c}$ is the number of valid positive samples for the c‐th observation label, $m_{i,c}$ ensures that invalid annotations are not counted and I(·) is the indicator function. This definition computes the class‐frequency prior from certain positive labels and avoids mixing the soft target value of uncertain labels into the positive sample count. The class‐frequency weight is first computed as:

(9) $$\;{\hat{\alpha }}_{c}=\frac{1-\beta }{1-\beta^{n_{c}}}\;,\;0\leq \beta <1.$$

where, ${\hat{\alpha }}_{c}$ is the unnormalized frequency‐based weight for the c‐th observation label, $n_{c}$ is the number of valid positive samples for this label, and β controls the degree of class balancing. A rarer observation label receives a larger ${\hat{\alpha }}_{c}$.

To keep the average weight stable, the normalized class weight is defined as:

(10) $$\;\alpha_{c}=\frac{C{\hat{\alpha }}_{c}}{\sum_{k=1}^{C}{\hat{\alpha }}_{k}}\;.$$

where, $\alpha_{c}$ denotes the normalized class‐frequency weight used in the final sample‐label weight, C is the number of observation labels, and the denominator sums the unnormalized weights over all labels. This normalization preserves relative class balancing while avoiding an overall change in the loss scale. The uncertainty suppression factor is then defined as:

(11) $$s_{i,c}=\max\left(\varepsilon ,1-\lambda u_{i,c}\right),\;0<\varepsilon \leq 1,\;0\leq \lambda \leq 1.$$

where, $s_{i,c}$ is the suppression factor, $u_{i,c}$ is the uncertainty value of the annotation, λ controls the suppression strength, and ε prevents the weight from becoming zero. When the label is certain, $u_{i,c}=0$ and $s_{i,c}=1$. When the label is uncertain, $u_{i,c}=1$ and $s_{i,c}$ is reduced to max(ε,1−λ). The final sample‐label weight is

(12) $$\;\omega_{i,c}=m_{i,c}\;\alpha_{c}s_{i,c}.$$

where, $\omega_{i,c}$ is the final sample‐label weight. The term $m_{i,c}$ removes invalid labels, $\alpha_{c}$ increases the contribution of rare labels, and $s_{i,c}$ suppresses uncertain annotations. Thus, tail‐class supervision is enhanced only when the annotation is sufficiently reliable. As illustrated in Figure 2, this weighting mechanism enhances certain tail‐class supervision while suppressing the excessive contribution of uncertain tail‐class annotations.

**FIGURE 2:**
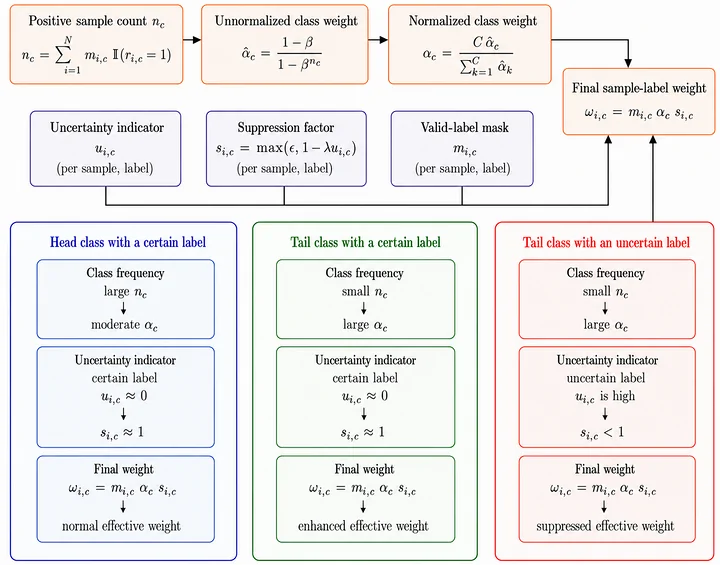
Illustration of uncertainty‐suppressed long‐tailed weighting for head classes, certain tail‐class labels, and uncertain tail‐class labels.

The proposed training loss is finally written as

(13) $$\;L_{\mathrm{USLT}}=\frac{1}{\sum_{i=1}^{N}\sum_{c=1}^{C}m_{i,c}}\;\sum_{i=1}^{N}\sum_{c=1}^{C}\omega_{i,c}\ell_{i,c}.$$

where, $L_{\mathrm{USLT}}$ is the final loss, $\omega_{i,c}$ is the sample‐label weight in Equation (12), and $\ell_{i,c}$ is the binary loss in Equation (7). During optimization, gradients are propagated through the encoder and the label‐adaptive head according to this loss. During inference, only the probabilities $p_{i,c}$ are used. The class‐frequency weights and uncertainty factors are not required.

The loss differs from ordinary class‐balanced learning in the way it treats rare but uncertain labels. A frequency‐only strategy increases the effect of every tail‐class annotation, while the proposed strategy increases the effect of a tail class only after considering its annotation state. This is important for report‐mined CXR datasets, where rare labels may contain more ambiguous descriptions or incomplete findings. The suppression factor does not remove uncertain samples from training. It only reduces their dominance after class balancing, which keeps useful weak supervision while limiting the influence of unreliable labels. The overall method remains compact because the label‐adaptive head adds only a small number of parameters through the bottleneck adapter and label‐conditioned gates, and the loss introduces no additional network branch. As a result, USLFA can be trained using the same pipeline as common multi‐label classifiers and remains compatible with different visual encoders.

## 4 EXPERIMENTS

### 4.1 Dataset and evaluation protocol

The primary experiments use CheXpert, which contains 224,316 chest radiographs from 65,240 patients and provides report‐derived labels for 14 observations.^4^ The publicly released training and validation files are merged and repartitioned at the patient level into 70% training, 10% validation, and 20% held‐out test subsets. All images from one patient remain in the same subset, and the same primary split is used for the main comparisons. Two additional patient‐level partitions with the same 70%/10%/20% ratio are generated only for split‐sensitivity analysis. Images are resized to 224 × 224 pixels. To complement the custom split, the primary‐split models are also evaluated on the official CheXpert test set, which contains 500 studies from 500 unseen patients.^4^ The reference standard is based on majority‐vote radiologist annotations for the five competition observations: Atelectasis, Cardiomegaly, Consolidation, Edema, and Pleural Effusion. The official test set is used only for final evaluation and does not contribute to model training, hyperparameter selection, uncertain‐label strategy selection, or threshold fitting.

MIMIC‐CXR‐JPG is used for independent cross‐dataset evaluation.^5^ MIMIC‐CXR‐JPG contains 377,110 radiographs from 65,379 patients, with structured labels available for 227,827 studies. We use its recommended test split, comprising 5,159 images from 3,269 studies of 293 patients. Models trained on the primary CheXpert split are transferred without retraining or fine‐tuning. The 14‐label space is retained, label‐wise F1 thresholds remain those selected on the CheXpert validation subset, and the CheXpert‐defined head, medium, and tail groups are kept fixed. Because MIMIC‐CXR‐JPG provides study‐level labels, predictions from multiple radiographs belonging to the same study are aggregated before evaluation. For each observation, the study‐level prediction is defined as the maximum predicted probability across all available views within that study, following the multi‐view aggregation strategy used in the original CheXpert evaluation. The same aggregation rule is applied to all compared methods.

In the main protocol, positive labels are mapped to 1, negative and blank labels are mapped to 0, and uncertain labels are assigned a soft target of 0.5. For USLFA, uncertain labels have the uncertainty indicator set to 1 and remain valid in the loss, whereas the valid‐label mask is used to implement ignore‐based comparison strategies. Other uncertain‐label handling strategies are implemented according to the definitions in Section 4.F. The above label conversion applies to model training. During evaluation, uncertain ground‐truth entries are excluded from the calculation of AUROC, AUPRC, and F1 for the corresponding observation rather than being forced into either binary class. Positive labels are evaluated as 1, whereas negative and blank labels are evaluated as 0. The same evaluation convention is applied to the custom CheXpert test subsets and MIMIC‐CXR‐JPG.

Table 1 shows that a few common labels account for most positive observations, whereas rare labels have substantially fewer samples. The head, medium, and tail groups are determined by ranking the positive counts of all labels. This grouping is used only for analysis and does not change the training process. The positive counts in Table 1 summarize the certain positive labels in the publicly available CheXpert annotations and are used to describe the long‐tailed label distribution. In training, class‐frequency weights are computed only from the training subset of the custom patient‐level split to avoid using validation or test information. Uncertain labels are not counted as positive samples when estimating $n_{c}$. According to the ranked positive counts, the top four labels are assigned to the head group, the next five labels to the medium group, and the remaining five labels to the tail group.

**TABLE 1:** Statistics of the CheXpert label distribution used in the experiments.

Label	Positive count	Share (%)	Group
Support Devices	116001	22.50	Head
Lung Opacity	105581	20.48	Head
Pleural Effusion	86187	16.72	Head
Edema	52246	10.13	Head
Atelectasis	33376	6.47	Medium
Cardiomegaly	27000	5.24	Medium
No Finding	22381	4.34	Medium
Pneumothorax	19448	3.77	Medium
Consolidation	14783	2.87	Medium
Enlarged Cardiomediastinum	10798	2.09	Tail
Lung Lesion	9186	1.78	Tail
Fracture	9040	1.75	Tail
Pneumonia	6039	1.17	Tail
Pleural Other	3523	0.68	Tail

The area under the receiver operating characteristic curve (AUROC) and the area under the precision‐recall curve (AUPRC) are computed independently for each label and then macro‐averaged over the 14 observation labels. Macro F1 is also reported to evaluate threshold‐dependent multi‐label recognition performance under class imbalance. Macro‐AUPRC is designated as the primary endpoint because precision‐recall analysis is more informative than receiver operating characteristic analysis when the positive class is relatively rare.^46^ Macro‐AUROC and macro F1 are treated as secondary endpoints. Tail‐label AUPRC is included as a designated group‐wise endpoint for evaluating rare‐label recognition. For the head, medium, and tail analyses, the group‐level results are calculated by averaging the corresponding label‐wise metrics within each frequency group.

(14) $$F\;1_{c}=\frac{2P_{c}R_{c}}{P_{c}+R_{c}}\;.$$

where, $P_{c}$ and $R_{c}$ denote the precision and recall for the c‐th label. Macro F1 is denoted by $\bar{F1}$ and is obtained by averaging the label‐wise F1 scores as

(15) $$\;\bar{F1}=\frac{1}{C}\;\sum_{c=1}^{C}F1_{c}.$$

where, C denotes the number of evaluated observation labels. For the head, medium, and tail analysis, the group level result of a metric M is computed as

(16) $$\;M_{G}=\frac{1}{\left|G\right|}\;\sum_{c\in G}M_{c}.$$

where, G denotes one label group, |G| is the number of labels in the group, and $M_{c}$ is the per label metric value for the c‐th label.

### 4.2 Implementation details

DenseNet‐121^47^ and ConvNeXt‐Tiny^48^ are used as representative visual backbones. DenseNet‐121 is included as a commonly used CXR classification baseline,^35^, ^36^ while ConvNeXt‐Tiny provides a modern convolutional backbone with stronger representation capacity.^48^ All backbones are initialized with ImageNet‐pretrained weights,^49^ and grayscale CXR images are converted into three‐channel inputs by channel replication. The final classification layer is replaced by a 14‐label multi‐label classifier.

Images are resized to 224 × 224 pixels and normalized using ImageNet statistics.^49^ During training, random horizontal flipping with a probability of 0.5 and random resized cropping are applied. All models are implemented in PyTorch^50^ and trained on an NVIDIA GeForce RTX 4090 graphics processing unit (GPU) using AdamW^51^ for 30 epochs, with a batch size of 32, an initial learning rate of 1 × 10^−4^, a weight decay of 1 × 10^−4^, and a cosine learning‐rate schedule^52^ with five warm‐up epochs.

For macro F1, label‐wise decision thresholds are selected on the validation set by maximizing the F1 score of each label and are then fixed before test‐set evaluation. The thresholds are not re‐estimated during bootstrap resampling or permutation testing. For USLFA, the adapter hidden dimension is set to 256 and the class‐balanced parameter β is set to 0.999. The uncertainty suppression coefficient λ is evaluated at 0, 0.25, 0.50, 0.75, and 1.00 on the validation subset, and the final value is selected according to validation macro‐AUPRC before test evaluation. For ASL, CBL, and LDAM, U Zeros, U Ones, U Ignore, and U Soft are likewise compared on the validation subset, and the best strategy for each loss is fixed before held‐out test evaluation. All methods otherwise use the same preprocessing, optimizer, learning‐rate schedule, and validation‐based threshold selection procedure.

### 4.3 Statistical analysis

Statistical analyses were performed at the patient level to account for the correlation among multiple radiographs from the same patient. Ninety‐five percent confidence intervals (CIs) for macro‐AUROC, macro‐AUPRC, macro F1, group‐wise metrics, and between‐method differences were estimated using nonparametric patient‐level bootstrap resampling with 5,000 iterations. In each bootstrap iteration, patients were sampled with replacement, and all radiographs belonging to each selected patient were retained as a cluster. For paired comparisons, the same bootstrap sample was applied to both methods, and the 95% CI of the performance difference was obtained from the 2.5th and 97.5th percentiles of the paired bootstrap distribution.

Formal pairwise comparisons reported in Tables 2, 3, 4, 5 were performed using patient‐level paired permutation tests with 10,000 permutations. Prediction outputs from the two compared methods were paired by patient. During each permutation, the complete prediction outputs of the two methods were exchanged within each patient with a probability of 0.5, after which the difference in the corresponding performance metric was recalculated. All tests were two‐sided. Macro‐AUPRC was designated as the primary endpoint, whereas macro‐AUROC and macro F1 were treated as secondary endpoints. Tail‐label AUPRC was included as a designated group‐wise endpoint for rare‐label recognition. The Holm procedure was applied to the formal pairwise comparisons in the primary CheXpert analyses reported in Tables 2, 3, 4, 5 to control the family‐wise error rate. A Holm‐adjusted p value below 0.05 was considered statistically significant.

**TABLE 2:** Overall performance comparison on the held‐out CheXpert test subset. Values are reported with patient‐level bootstrap 95% CIs.

Method	AUROC (95% CI)	AUPRC (95% CI)	Macro F1 (95% CI)
D+BCE	0.818 (0.810–0.825)	0.472 (0.455–0.486)	0.426 (0.411–0.440)
D+ASL	0.826 (0.817–0.834)	0.486 (0.471–0.501)	0.438 (0.422–0.452)
C+BCE	0.836 (0.828–0.843)	0.496 (0.481–0.509)	0.450 (0.436–0.463)
C+ASL	0.844 (0.835–0.852)	0.512 (0.496–0.526)	0.466 (0.451–0.480)
C+CBL	0.848 (0.840–0.856)	0.521 (0.504–0.535)	0.475 (0.459–0.489)
C+LDAM	0.852 (0.843–0.860)	0.529 (0.513–0.543)	0.484 (0.468–0.498)
USLFA	0.864 (0.856–0.871)	0.547 (0.532–0.560)	0.501 (0.487–0.514)

*Note*. D denotes DenseNet‐121, and C denotes ConvNeXt‐Tiny. AUROC, area under the receiver operating characteristic curve; AUPRC, area under the precision‐recall curve; BCE, binary cross‐entropy; ASL, asymmetric loss; CBL, class‐balanced loss; LDAM, label‐distribution‐aware margin.

**TABLE 3:** Group‐wise performance on head, medium, and tail labels. Values are reported with patient‐level bootstrap 95% CIs.

Method	Head group	Medium group	Tail group
BCE	AUROC 0.917 (0.907–0.925)	AUROC 0.876 (0.864–0.887)	AUROC 0.731 (0.701–0.758)
	AUPRC 0.740 (0.721–0.757)	AUPRC 0.582 (0.560–0.603)	AUPRC 0.214 (0.181–0.245)
	F1 0.640 (0.622–0.657)	F1 0.501 (0.478–0.522)	F1 0.247 (0.215–0.278)
ASL	AUROC 0.915 (0.905–0.923)	AUROC 0.873 (0.861–0.884)	AUROC 0.758 (0.728–0.785)
	AUPRC 0.737 (0.718–0.755)	AUPRC 0.597 (0.575–0.617)	AUPRC 0.247 (0.212–0.280)
	F1 0.635 (0.617–0.652)	F1 0.510 (0.488–0.531)	F1 0.286 (0.252–0.318)
CBL	AUROC 0.911 (0.901–0.920)	AUROC 0.873 (0.860–0.885)	AUROC 0.773 (0.744–0.800)
	AUPRC 0.728 (0.708–0.746)	AUPRC 0.606 (0.584–0.626)	AUPRC 0.270 (0.234–0.304)
	F1 0.628 (0.609–0.646)	F1 0.523 (0.500–0.544)	F1 0.304 (0.270–0.337)
LDAM	AUROC 0.914 (0.904–0.922)	AUROC 0.868 (0.855–0.880)	AUROC 0.786 (0.757–0.813)
	AUPRC 0.733 (0.714–0.751)	AUPRC 0.608 (0.586–0.629)	AUPRC 0.286 (0.250–0.320)
	F1 0.633 (0.615–0.651)	F1 0.524 (0.502–0.545)	F1 0.324 (0.289–0.357)
USLFA	AUROC 0.919 (0.909–0.927)	AUROC 0.872 (0.859–0.884)	AUROC 0.812 (0.783–0.839)
	AUPRC 0.746 (0.727–0.763)	AUPRC 0.603 (0.581–0.623)	AUPRC 0.331 (0.294–0.366)
	F1 0.649 (0.631–0.666)	F1 0.512 (0.489–0.533)	F1 0.371 (0.335–0.405)

*Note*. All methods use ConvNeXt‐Tiny. AUROC, area under the receiver operating characteristic curve; AUPRC, area under the precision‐recall curve; F1, F1 score; BCE, binary cross‐entropy; ASL, asymmetric loss; CBL, class‐balanced loss; LDAM, label‐distribution‐aware margin.

**TABLE 4:** Ablation study of the proposed components. Values are reported with patient‐level bootstrap 95% CIs.

Setting	LFA	LT	US	AUROC (95% CI)	AUPRC (95% CI)	Macro F1 (95% CI)	Tail AUPRC (95% CI)
Base	0	0	0	0.836 (0.828–0.843)	0.496 (0.481–0.509)	0.450 (0.436–0.463)	0.214 (0.181–0.245)
+LFA	1	0	0	0.845 (0.837–0.852)	0.515 (0.500–0.529)	0.466 (0.452–0.479)	0.239 (0.205–0.272)
+LT	1	1	0	0.854 (0.846–0.861)	0.532 (0.517–0.546)	0.486 (0.472–0.499)	0.298 (0.262–0.333)
+US	1	0	1	0.849 (0.841–0.856)	0.523 (0.508–0.537)	0.476 (0.462–0.489)	0.264 (0.229–0.298)
Full	1	1	1	0.864 (0.856–0.871)	0.547 (0.532–0.560)	0.501 (0.487–0.514)	0.331 (0.294–0.366)

*Note*. LFA, lightweight feature adaptation; LT, long‐tailed re‐weighting; US, uncertainty suppression; AUROC, area under the receiver operating characteristic curve; AUPRC, area under the precision‐recall curve.

**TABLE 5:** Comparison of uncertain‐label handling strategies. Values are reported with patient‐level bootstrap 95% CIs.

Strategy	AUROC (95% CI)	AUPRC (95% CI)	Macro F1 (95% CI)	Tail AUPRC (95% CI)
U Zeros	0.846 (0.838–0.853)	0.520 (0.505–0.534)	0.475 (0.461–0.488)	0.278 (0.242–0.312)
U Ones	0.850 (0.842–0.857)	0.527 (0.512–0.541)	0.482 (0.468–0.495)	0.293 (0.257–0.328)
U Ignore	0.842 (0.834–0.849)	0.511 (0.496–0.525)	0.467 (0.453–0.480)	0.263 (0.227–0.297)
U Soft	0.853 (0.845–0.860)	0.535 (0.520–0.549)	0.489 (0.475–0.502)	0.306 (0.270–0.341)
USLFA	0.864 (0.856–0.871)	0.547 (0.532–0.560)	0.501 (0.487–0.514)	0.331 (0.294–0.366)

*Note*. U Zeros maps uncertain labels to 0; U Ones maps uncertain labels to 1; U Ignore excludes uncertain labels from the loss; U Soft assigns a soft target of 0.5. AUROC, area under the receiver operating characteristic curve; AUPRC, area under the precision‐recall curve.

Label‐wise decision thresholds were determined exclusively from the validation subset and remained fixed during test‐set bootstrap resampling and permutation testing. The feature‐head compatibility analysis, validation‐based uncertain‐label strategy selection, λ sensitivity analysis, and repeated‐split analysis were treated as supporting analyses and interpreted descriptively. For MIMIC‐CXR‐JPG and the official CheXpert test set, 95% CIs were estimated using 5,000 patient‐level bootstrap samples, and no additional family of pairwise significance tests was introduced.

### 4.4 Main comparison results

For fair comparison, all ConvNeXt‐Tiny baselines use the same patient‐level data split, preprocessing, optimizer, batch size, learning‐rate schedule, and validation‐based threshold selection procedure. The compared loss functions include BCE, ASL,^23^ CBL,^21^ and LDAM loss.^22^ For CBL, the effective‐number parameter β is set to 0.999, consistent with the setting used in USLFA. For LDAM loss, the class‐frequency‐dependent margin is applied independently to each binary label classification task. To avoid favoring USLFA through a particular uncertain‐label conversion, U Zeros, U Ones, U Ignore, and U Soft are screened separately for ASL, CBL, and LDAM on the validation subset. The selected strategy for each loss is fixed before held‐out test evaluation and is reported in Section 4.G. The resulting validation‐selected configuration is used in the corresponding main comparison, while all other training and evaluation settings remain unchanged.

Table 2 shows that USLFA achieves a macro‐AUROC of 0.864 (95% CI, 0.856‐0.871), a macro‐AUPRC of 0.547 (95% CI, 0.532‐0.560), and a macro F1 of 0.501 (95% CI, 0.487‐0.514). Compared with C+BCE, the paired differences are 0.028 for macro‐AUROC (95% CI, 0.018‐0.038; Holm‐adjusted *p <* 0.001), 0.051 for macro‐AUPRC (95% CI, 0.036‐0.066; Holm‐adjusted *p <* 0.001), and 0.051 for macro F1 (95% CI, 0.034‐0.068; Holm‐adjusted *p <* 0.001). Compared with C+LDAM, USLFA improves macro‐AUROC by 0.012 (95% CI, 0.003‐0.021; Holm‐adjusted *p =* 0.013), macro‐AUPRC by 0.018 (95% CI, 0.003–0.033; Holm‐adjusted *p =* 0.024), and macro F1 by 0.017 (95% CI, 0.001‐0.033; Holm‐adjusted *p =* 0.041). The larger absolute differences in macro‐AUPRC and macro F1 indicate that the principal benefit of USLFA is associated with positive‐label recognition under class imbalance rather than only with ranking performance.

Figure 3 presents the receiver operating characteristic and precision‐recall curves for representative head, medium, and tail labels. For Pleural Effusion, the curves of the compared methods remain relatively close, which is consistent with the limited differences observed for frequent labels. For Pneumothorax and Pleural Other, USLFA maintains numerically higher precision across a wider recall range. Because these curves represent selected labels, formal conclusions are based on the patient‐level statistical comparisons rather than visual inspection alone. The curve patterns are nevertheless consistent with the group‐wise results reported in Table 3.

**FIGURE 3:**
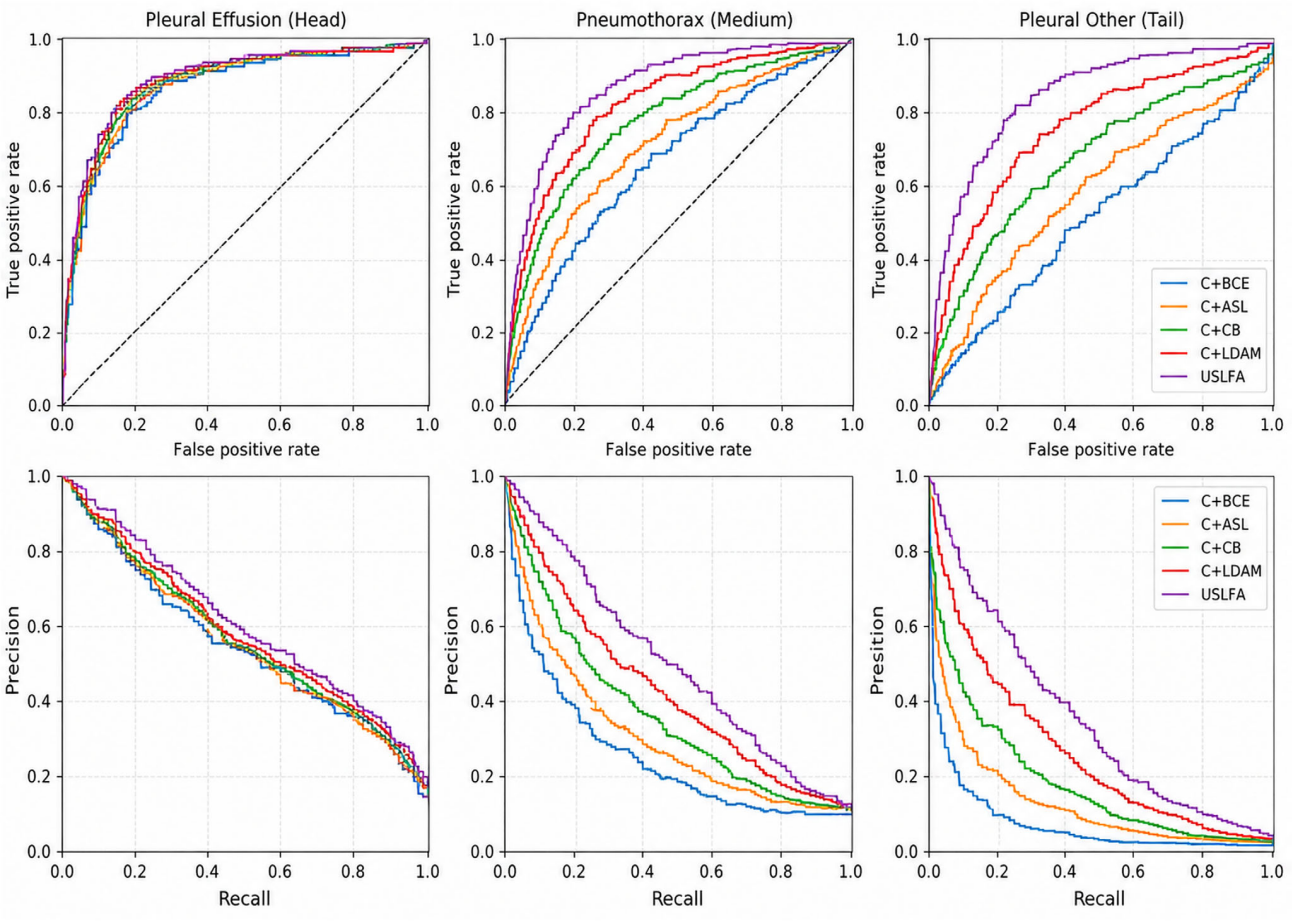
Receiver operating characteristic (ROC) and precision‐recall curves for representative head, medium, and tail labels. The top row shows ROC curves, and the bottom row shows precision‐recall curves. The three columns correspond to Pleural Effusion, Pneumothorax, and Pleural Other, respectively. Curves are computed using prediction scores from the held‐out test subset.

### 4.5 Head, medium, and tail label analysis

To examine whether the improvement is concentrated on rare labels or shared across different frequency groups, we further report group‐wise AUROC, AUPRC, and F1 for head, medium, and tail labels.

Table 3 provides a more detailed analysis across label‐frequency groups. For the tail group, USLFA achieves an AUPRC of 0.331 (95% CI, 0.294‐0.366), compared with 0.214 (95% CI, 0.181‐0.245) for BCE and 0.286 (95% CI, 0.250‐0.320) for LDAM. The paired tail‐AUPRC difference is 0.117 versus BCE (95% CI, 0.078‐0.157; Holm‐adjusted *p* < 0.001) and 0.045 versus LDAM (95% CI, 0.012‐0.079; Holm‐adjusted *p* = 0.018). In comparison, head‐label AUPRC changes only from 0.740 (95% CI, 0.721‐0.757) with BCE to 0.746 (95% CI, 0.727‐0.763) with USLFA, whereas medium‐label AUPRC increases from 0.582 (95% CI, 0.560‐0.603) to 0.603 (95% CI, 0.581‐0.623). This pattern indicates that the improvement is more pronounced for medium‐ and tail‐frequency labels than for frequent labels.

Figure 4 provides an exploratory per‐label analysis of representative tail categories. USLFA produces higher AUPRC point estimates than BCE and frequency‐only long‐tailed weighting for all displayed labels. The size of the improvement varies with the label frequency and uncertain‐label proportion, indicating that tail labels are not uniform in either prevalence or annotation reliability. Because the per‐label comparisons are exploratory and were not individually included in the formal multiple‐testing procedure, they are interpreted descriptively. Formal statistical conclusions are based on the macro‐ and group‐level comparisons reported in Tables 2 and 3.

**FIGURE 4:**
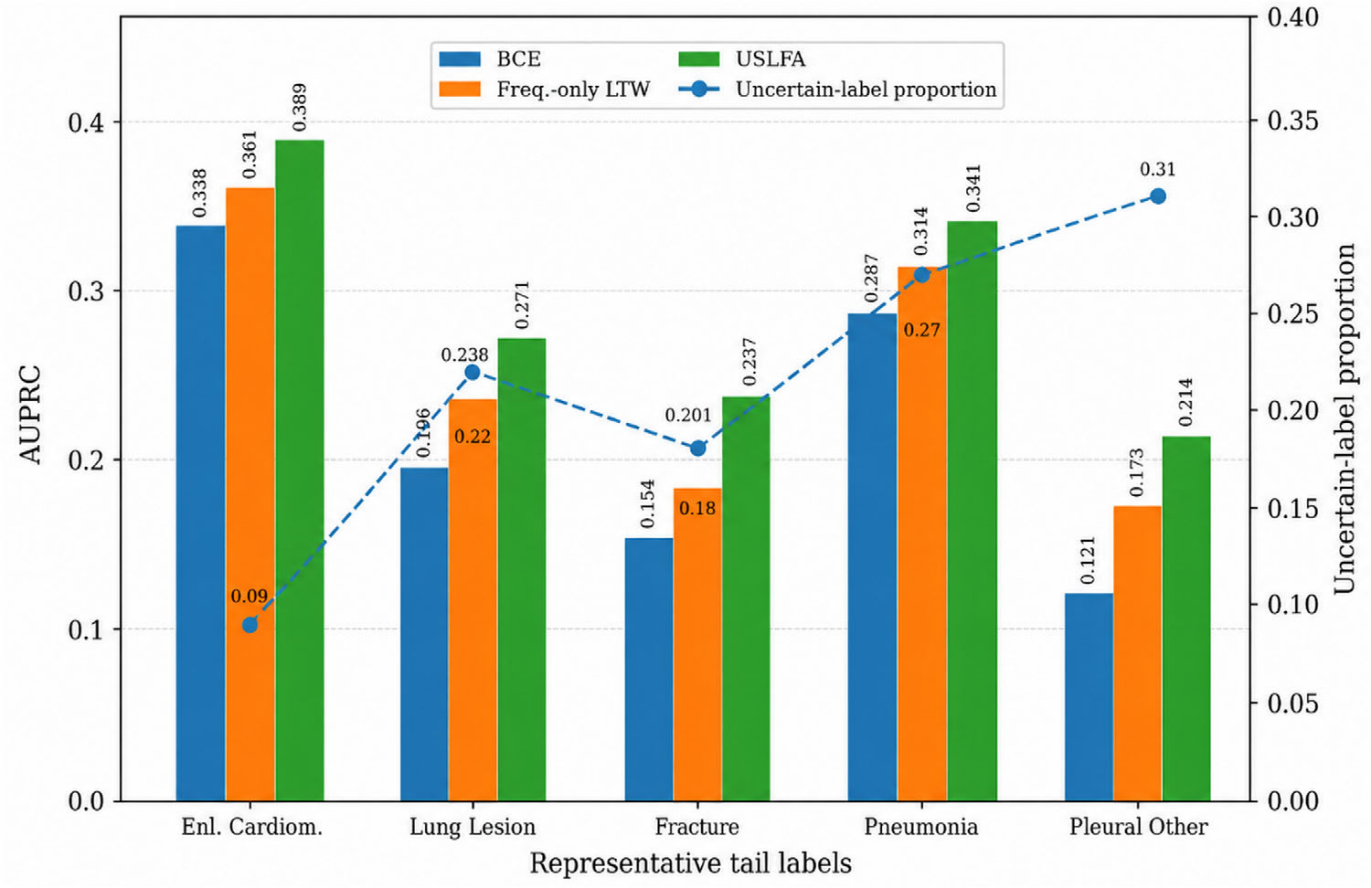
Per‐label area under the precision‐recall curve (AUPRC) for representative tail labels and the corresponding uncertain‐label proportions. Bars show the point estimates, and error bars denote patient‐level bootstrap 95% CIs. BCE denotes binary cross‐entropy. The dashed line denotes the uncertain‐label proportion of each observation label.

To further examine whether the label‐wise improvements in Figure 4 are reflected in visual evidence, Grad‐CAM visualizations^44^ are provided in Figure 5 for representative tail‐label predictions. Each row presents the original CXR image and the activation maps generated by BCE, frequency‐only long‐tailed weighting, and USLFA. Compared with BCE and frequency‐only weighting, USLFA tends to produce less scattered activation patterns in the selected examples. These qualitative examples are consistent with the quantitative results in Table 3, Table 5, and Figure 4, but they are used only as supplementary visual evidence because Grad‐CAM does not provide lesion‐level ground truth.

**FIGURE 5:**
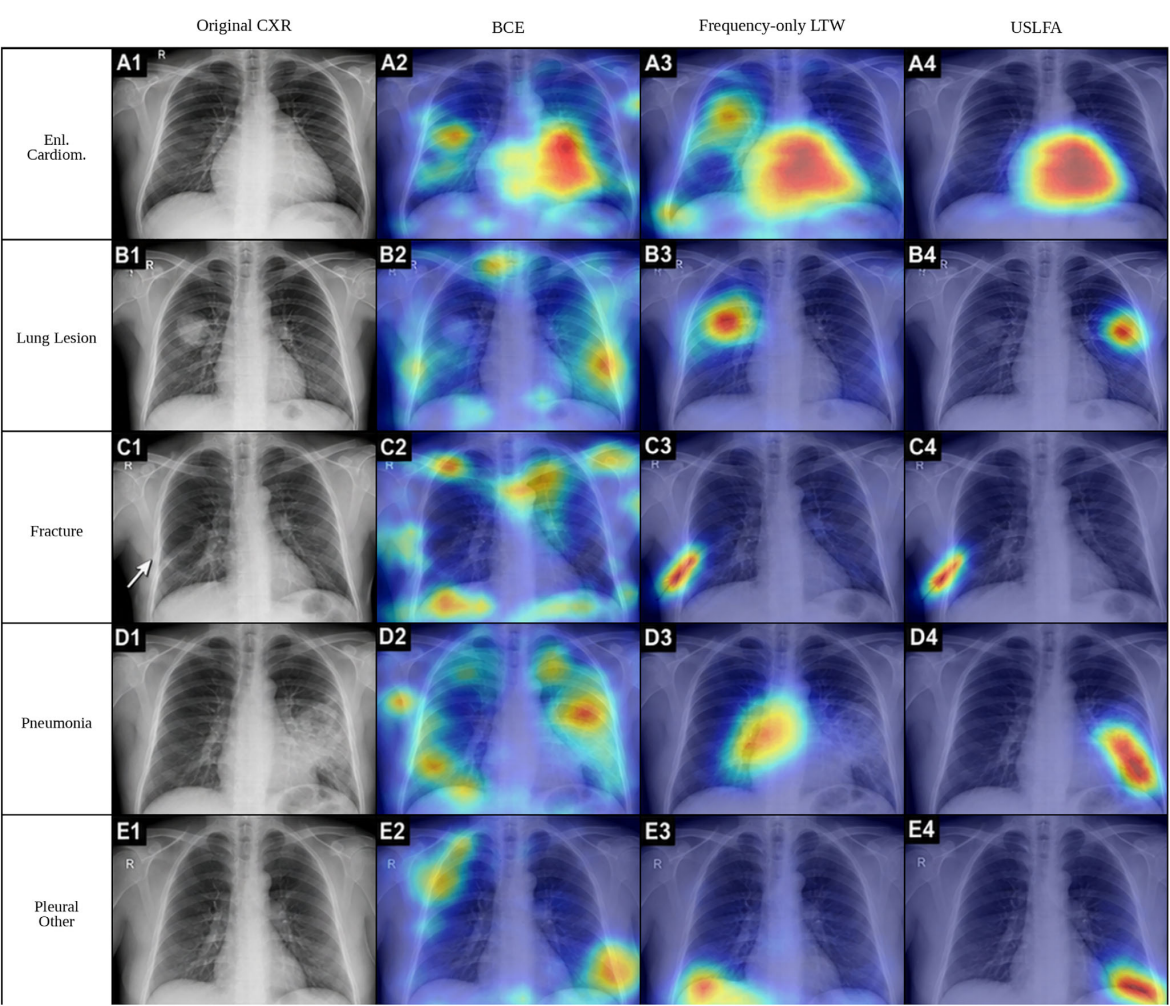
Grad‐CAM visualization of representative tail‐label predictions.

### 4.6 Ablation and component analysis

The ablation study is conducted to separate the effects of the lightweight feature adaptation (LFA) module, the frequency‐based long‐tailed re‐weighting (LT), and the uncertainty suppression (US) factor.

Table 4 evaluates the contribution of lightweight feature adaptation, long‐tailed re‐weighting, and uncertainty suppression. Adding the lightweight label‐adaptive feature head increases macro‐AUPRC from 0.496 to 0.515 and tail‐label AUPRC from 0.214 to 0.239. Introducing frequency‐based long‐tailed re‐weighting further increases tail‐label AUPRC to 0.298, whereas applying uncertainty suppression without long‐tailed re‐weighting produces a tail‐label AUPRC of 0.264. The full USLFA model achieves a macro‐AUPRC of 0.547 and a tail‐label AUPRC of 0.331. Compared with the +LT configuration, the full model increases macro‐AUPRC by 0.015 (95% CI, 0.001‐0.030; Holm‐adjusted *p =* 0.046) and tail‐label AUPRC by 0.033 (95% CI, 0.004‐0.063; Holm‐adjusted *p =* 0.032). Compared with the +US configuration, the corresponding differences are 0.024 for macro‐AUPRC (95% CI, 0.009‐0.039; Holm‐adjusted *p =* 0.006) and 0.067 for tail‐label AUPRC (95% CI, 0.033‐0.102; Holm‐adjusted *p =* 0.001). These results support the complementary roles of frequency‐based re‐weighting and uncertainty suppression.

To isolate the effect of uncertain‐label handling, all strategies in Table 5 use the same ConvNeXt‐Tiny backbone, the same lightweight label‐adaptive feature head, the same frequency‐based long‐tailed re‐weighting, and the same training protocol. The only difference lies in how uncertain labels are converted or weighted during optimization. U Zeros maps uncertain labels to 0, U Ones maps them to 1, U Ignore removes them from the loss by setting the corresponding valid‐label mask to 0, and U Soft assigns uncertain labels a soft target of 0.5 without uncertainty suppression. USLFA also assigns uncertain labels a soft target of 0.5, but further applies the uncertainty suppression factor to reduce their effective weights after long‐tailed re‐weighting.

Table 5 compares alternative treatments of uncertain labels under the same backbone, feature head, and long‐tailed weighting scheme. U Soft provides the most direct reference because it retains uncertain labels as soft targets without applying uncertainty suppression. USLFA increases macro‐AUPRC from 0.535 (95% CI, 0.520‐0.549) to 0.547 (95% CI, 0.532‐0.560) and tail‐label AUPRC from 0.306 (95% CI, 0.270‐0.341) to 0.331 (95% CI, 0.294‐0.366). The corresponding differences remain significant after Holm correction (Holm‐adjusted *p =* 0.048 and *p =* 0.045, respectively). Macro‐AUROC also improves significantly (Holm‐adjusted *p =* 0.021), whereas the additional macro F1 gain over U Soft does not remain significant after Holm correction (*p =* 0.052). These results indicate that uncertainty suppression mainly strengthens ranking and rare‐label recognition beyond soft‐label conversion alone.

To further distinguish the contribution of the feature head from that of the loss function, LFA is combined separately with BCE, ASL, CBL, and LDAM while all other settings are kept unchanged. Within each paired comparison, the uncertain‐label handling strategy is held fixed. For ASL, CBL, and LDAM, the strategy follows the validation‐selected setting reported in Table 7, whereas BCE uses the same main‐protocol uncertain‐label setting with and without LFA. Thus, the only architectural difference within each pair is the inclusion of LFA. The results are reported in Table 6.

**TABLE 6:** Effect of lightweight feature adaptation across different loss functions. Values are reported with patient‐level bootstrap 95% CIs.

Loss	LFA	AUROC (95% CI)	AUPRC (95% CI)	Macro F1 (95% CI)	Tail AUPRC (95% CI)
BCE	×	0.836 (0.828–0.843)	0.496 (0.481–0.509)	0.450 (0.436–0.463)	0.214 (0.181–0.245)
BCE	√	0.845 (0.837–0.852)	0.515 (0.500–0.529)	0.466 (0.452–0.479)	0.239 (0.205–0.272)
ASL	×	0.844 (0.835–0.852)	0.512 (0.496–0.526)	0.466 (0.451–0.480)	0.247 (0.212–0.280)
ASL	√	0.850 (0.841–0.858)	0.524 (0.508–0.539)	0.476 (0.460–0.490)	0.265 (0.229–0.300)
CBL	×	0.848 (0.840–0.856)	0.521 (0.504–0.535)	0.475 (0.459–0.489)	0.270 (0.234–0.304)
CBL	√	0.857 (0.848–0.865)	0.535 (0.518–0.550)	0.489 (0.473–0.503)	0.295 (0.258–0.330)
LDAM	×	0.852 (0.843–0.860)	0.529 (0.513–0.543)	0.484 (0.468–0.498)	0.286 (0.250–0.320)
LDAM	√	0.859 (0.850–0.867)	0.539 (0.523–0.553)	0.493 (0.477–0.507)	0.303 (0.267–0.337)

*Note*. All methods use ConvNeXt‐Tiny. BCE, binary cross‐entropy; ASL, asymmetric loss; CBL, class‐balanced loss; LDAM, label‐distribution‐aware margin; LFA, lightweight feature adaptation.

Table 6 shows that the contribution of LFA is retained across different loss functions. Macro‐AUPRC increases from 0.496 to 0.515 with BCE, from 0.512 to 0.524 with ASL, from 0.521 to 0.535 with CBL, and from 0.529 to 0.539 with LDAM. Tail‐label AUPRC also improves for all four losses, with gains ranging from 0.017 to 0.025. The consistent direction of improvement indicates that the label‐adaptive feature head provides complementary feature adaptation rather than reproducing the effect of a particular long‐tailed loss.

### 4.7 Validation‐based fairness and sensitivity analyses

We first examine whether the main baseline comparison depends on the treatment of uncertain labels. For ASL, CBL, and LDAM, U Zeros, U Ones, U Ignore, and U Soft are evaluated independently on the CheXpert validation subset. Strategy selection is based exclusively on validation macro‐AUPRC, and the selected setting is fixed before held‐out test evaluation.

As shown in Table 7, U Soft yields the highest validation macro‐AUPRC for ASL, CBL, and LDAM, with values of 0.516, 0.524, and 0.530, respectively. The same validation‐selected strategy is therefore used for these losses in the held‐out test comparison. This result reduces the possibility that the performance difference in Table 2 is caused by assigning a weaker uncertain‐label treatment to the competing methods.

**TABLE 7:** Validation‐based selection of uncertain‐label strategies for the competing loss functions.

Loss	Strategy	Validation macro‐AUPRC (95% CI)	Selected
ASL	U Zeros	0.502 (0.484–0.519)	×
ASL	U Ones	0.509 (0.492–0.525)	×
ASL	U Ignore	0.497 (0.479–0.514)	×
ASL	U Soft	0.516 (0.499–0.532)	√
CBL	U Zeros	0.510 (0.492–0.527)	×
CBL	U Ones	0.518 (0.501–0.535)	×
CBL	U Ignore	0.506 (0.488–0.523)	×
CBL	U Soft	0.524 (0.507–0.540)	√
LDAM	U Zeros	0.519 (0.502–0.536)	×
LDAM	U Ones	0.526 (0.508–0.542)	×
LDAM	U Ignore	0.513 (0.495–0.530)	×
LDAM	U Soft	0.530 (0.513–0.547)	√

We next examine the sensitivity of USLFA to the uncertainty suppression coefficient λ. Five values spanning the range of Equation (11) are evaluated on the validation subset. Macro‐AUPRC is used for model selection, while tail‐label AUPRC is additionally reported to assess the response of rare labels.

Table 8 shows that performance improves as λ increases from 0 to 0.50, with validation macro‐AUPRC increasing from 0.533 to 0.546 and tail‐label AUPRC from 0.303 to 0.327. Both metrics decrease when stronger suppression is applied, reaching 0.536 and 0.311 at λ= 1.00. The validation results therefore support the selected value of λ= 0.50, suggesting that moderate suppression provides a better balance between retaining informative uncertain supervision and limiting its influence after class re‐weighting.

**TABLE 8:** Sensitivity to the uncertainty suppression coefficient λ on the CheXpert validation subset.

λ	Macro‐AUPRC (95% CI)	Tail AUPRC (95% CI)
0.00	0.533 (0.517–0.548)	0.303 (0.267–0.338)
0.25	0.541 (0.525–0.556)	0.318 (0.282–0.352)
0.50	0.546 (0.530–0.560)	0.327 (0.290–0.362)
0.75	0.542 (0.526–0.557)	0.321 (0.285–0.356)
1.00	0.536 (0.520–0.551)	0.311 (0.275–0.346)

### 4.8 Robustness and independent evaluation

To examine whether the main findings depend on a particular patient‐level partition, the comparison among C+BCE, C+LDAM, and USLFA is repeated on two additional 70%/10%/20% patient‐level splits. Each partition uses training‐derived class frequencies and validation‐derived decision thresholds independently, while the remaining training settings are unchanged.

Table 9 shows that the relative ordering of the three methods is preserved across all patient‐level partitions. USLFA achieves macro‐AUPRC values of 0.547, 0.540, and 0.555 on the primary, Split B, and Split C partitions, respectively, compared with 0.529, 0.524, and 0.534 for LDAM. The corresponding tail‐label AUPRC values for USLFA are 0.331, 0.319, and 0.342. These results indicate that the observed improvement is not confined to the primary random partition, although its magnitude varies modestly across splits.

**TABLE 9:** Sensitivity to patient‐level data partitioning on CheXpert. Values are reported with patient‐level bootstrap 95% CIs.

Split	Method	AUROC (95% CI)	AUPRC (95% CI)	Macro F1 (95% CI)	Tail AUPRC (95% CI)
Primary	C+BCE	0.836 (0.828–0.843)	0.496 (0.481–0.509)	0.450 (0.436–0.463)	0.214 (0.181–0.245)
Primary	C+LDAM	0.852 (0.843–0.860)	0.529 (0.513–0.543)	0.484 (0.468–0.498)	0.286 (0.250–0.320)
Primary	USLFA	0.864 (0.856–0.871)	0.547 (0.532–0.560)	0.501 (0.487–0.514)	0.331 (0.294–0.366)
Split B	C+BCE	0.833 (0.825–0.841)	0.490 (0.475–0.504)	0.445 (0.431–0.459)	0.205 (0.173–0.236)
Split B	C+LDAM	0.849 (0.841–0.857)	0.524 (0.509–0.538)	0.478 (0.464–0.492)	0.279 (0.244–0.313)
Split B	USLFA	0.861 (0.853–0.869)	0.540 (0.525–0.554)	0.495 (0.481–0.508)	0.319 (0.283–0.353)
Split C	C+BCE	0.838 (0.830–0.846)	0.501 (0.486–0.515)	0.454 (0.440–0.467)	0.224 (0.191–0.256)
Split C	C+LDAM	0.854 (0.846–0.862)	0.534 (0.518–0.548)	0.489 (0.474–0.503)	0.293 (0.257–0.327)
Split C	USLFA	0.866 (0.858–0.874)	0.555 (0.540–0.569)	0.506 (0.492–0.519)	0.342 (0.305–0.377)

Cross‐dataset transfer is further evaluated on the recommended MIMIC‐CXR‐JPG test split using models trained on the primary CheXpert partition, without retraining, fine‐tuning, or threshold recalibration. The split contains 5,159 images from 3,269 studies of 293 patients. The original 14‐label space and CheXpert‐defined tail group are retained.

Table 10 shows that absolute performance decreases for all methods on MIMIC‐CXR‐JPG compared with the internal CheXpert evaluation, which is consistent with cross‐dataset distribution shift. USLFA nevertheless retains the highest point estimates, achieving a macro‐AUROC of 0.831 (95% CI, 0.820‐0.841), macro‐AUPRC of 0.485 (95% CI, 0.467‐0.502), and macro F1 of 0.447 (95% CI, 0.430‐0.464). Its CheXpert‐defined tail AUPRC reaches 0.250 (95% CI, 0.217‐0.283), compared with 0.220 (95% CI, 0.188‐0.252) for LDAM. Because no additional pairwise hypothesis‐testing family is introduced for this external analysis, these differences are interpreted as cross‐dataset point‐estimate comparisons rather than statistically significant superiority.

**TABLE 10:** Cross‐dataset evaluation on the MIMIC‐CXR‐JPG test split. Values are reported with patient‐level bootstrap 95% CIs.

Method	AUROC (95% CI)	AUPRC (95% CI)	Macro F1 (95% CI)	CheXpert‐defined tail AUPRC (95% CI)
C+BCE	0.801 (0.790–0.811)	0.437 (0.419–0.454)	0.406 (0.389–0.423)	0.174 (0.145–0.203)
C+ASL	0.809 (0.798–0.819)	0.450 (0.432–0.467)	0.417 (0.400–0.434)	0.191 (0.160–0.222)
C+CBL	0.813 (0.802–0.824)	0.458 (0.440–0.475)	0.425 (0.408–0.442)	0.207 (0.175–0.239)
C+LDAM	0.820 (0.809–0.830)	0.467 (0.449–0.484)	0.433 (0.416–0.450)	0.220 (0.188–0.252)
USLFA	0.831 (0.820–0.841)	0.485 (0.467–0.502)	0.447 (0.430–0.464)	0.250 (0.217–0.283)

Finally, the primary‐split models are evaluated on the official radiologist‐annotated CheXpert test set, which contains 500 studies from 500 previously unseen patients. Evaluation follows the five competition observations using the released radiologist majority‐vote reference standard. For studies with multiple radiographic views, the predicted probability for each observation is taken as the maximum probability across the available views, consistent with the original CheXpert evaluation protocol.

As shown in Table 11, USLFA achieves a mean AUROC of 0.920 (95% CI, 0.908‐0.931) on the official CheXpert test set, compared with 0.915 (95% CI, 0.903‐0.926) for LDAM and 0.907 (95% CI, 0.895‐0.918) for BCE. USLFA has higher AUROC point estimates than LDAM for Atelectasis, Cardiomegaly, Edema, and Pleural Effusion, while its Consolidation AUROC is slightly lower (0.892 versus 0.895). The overall mean therefore supports the performance trend observed on the custom CheXpert partitions without implying uniform improvement for every individual observation.

**TABLE 11:** AUROC on the official radiologist‐annotated CheXpert test set. Values are reported with patient‐level bootstrap 95% CIs.

Method	Atelectasis	Cardiomegaly	Consolidation	Edema	Pleural Effusion	Mean AUROC
C+BCE	0.874 (0.840–0.904)	0.908 (0.880–0.933)	0.887 (0.852–0.917)	0.919 (0.893–0.940)	0.947 (0.928–0.963)	0.907 (0.895–0.918)
C+LDAM	0.883 (0.850–0.911)	0.916 (0.889–0.939)	0.895 (0.861–0.924)	0.927 (0.902–0.947)	0.952 (0.933–0.967)	0.915 (0.903–0.926)
USLFA	0.890 (0.857–0.918)	0.923 (0.896–0.944)	0.892 (0.857–0.922)	0.936 (0.912–0.954)	0.958 (0.940–0.972)	0.920 (0.908–0.931)

## 5 DISCUSSION

This study addresses a practical problem in report‐mined chest radiograph classification, where class imbalance and annotation uncertainty occur simultaneously. Conventional long‐tailed learning increases the contribution of rare labels, but this strategy may also amplify unreliable gradients when rare observations have uncertain report‐derived states. USLFA combines a lightweight label‐adaptive feature head with uncertainty‐suppressed class re‐weighting and is intended as a task‐level training strategy rather than a replacement for large foundation encoders.

The patient‐level statistical analysis provides evidence that the observed improvements are not limited to isolated point estimates. Compared with the ConvNeXt‐Tiny BCE baseline, USLFA increases macro‐AUPRC by 0.051 and tail‐label AUPRC by 0.117, with both paired differences remaining statistically significant after Holm correction. Compared with the stronger LDAM baseline, the corresponding improvements are 0.018 and 0.045. In contrast, the change in head‐label AUPRC is relatively small, from 0.740 (95% CI, 0.721‐0.757) with BCE to 0.746 (95% CI, 0.727‐0.763) with USLFA, whereas medium‐label AUPRC increases from 0.582 (95% CI, 0.560‐0.603) to 0.603 (95% CI, 0.581‐0.623). This pattern suggests that the proposed method principally benefits medium‐ and tail‐frequency observations while maintaining stable recognition of frequent labels. The repeated‐split and independent evaluations reduce dependence on a single internal partition. Across three patient‐level splits, USLFA retains the highest macro‐AUPRC and tail‐label AUPRC. The same ordering is preserved when the primary models are transferred to MIMIC‐CXR‐JPG without retraining, and USLFA also attains the highest mean AUROC on the official radiologist‐annotated CheXpert test set. These analyses provide complementary evidence from repeated partitioning, an independent institution, and the radiologist majority‐vote reference standard of the official CheXpert test set, although they do not establish prospective clinical generalizability.

The ablation analysis further indicates that the lightweight feature head, long‐tailed re‐weighting, and uncertainty suppression address different aspects of the classification problem. Label‐adaptive feature transformation improves the shared representation, whereas long‐tailed re‐weighting strengthens rare‐label supervision. Uncertainty suppression provides an additional improvement when applied together with class‐frequency weighting, particularly for tail‐label AUPRC. The comparison with U Soft further indicates that assigning uncertain labels a target of 0.5 does not fully address the risk of amplifying uncertain rare‐label supervision. Explicitly coupling uncertainty information with the long‐tailed weight produces statistically significant improvements in macro‐AUROC, macro‐AUPRC, and tail‐label AUPRC, whereas the macro F1 difference does not remain significant after Holm correction. The stronger‐loss compatibility analysis provides a separate check on the contribution of the feature head. LFA improves macro‐AUPRC when combined with ASL, CBL, and LDAM, indicating that its effect is not specific to BCE. Validation‐based uncertain‐label strategy selection also confirms that the main comparison does not assign a weaker uncertainty treatment to the competing losses. The λ analysis shows a broad optimum at moderate suppression, which is consistent with the intended balance between retaining useful uncertain supervision and limiting its weight after class re‐weighting.

In a clinical workflow, USLFA changes model training rather than the inference interface. After training, the classifier outputs the same label probabilities as a standard multi‐label model and does not require uncertainty weights or an additional network branch at inference. The outputs could therefore be integrated into triage or decision‐support systems in the same form as conventional CXR classifiers. This study does not evaluate prospective clinical use, so the predictions should be interpreted as decision‐support scores rather than autonomous diagnoses.

Several limitations should be acknowledged. First, although the evaluation includes both the official CheXpert test set and MIMIC‐CXR‐JPG, external validation is limited to one additional institution, and the MIMIC labels remain report‐derived rather than a fully radiologist‐adjudicated image‐level reference. Second, the official CheXpert benchmark covers five competition observations and therefore does not validate the complete 14‐label setting. Third, report‐derived uncertainty states do not capture all forms of annotation error. Fourth, the Grad‐CAM analysis remains qualitative and does not provide lesion‐level localization validation. Finally, the study is retrospective and does not include prospective evaluation in a radiology information system or PACS workflow. Further work should include multi‐institutional and prospective validation.

## 6 CONCLUSION

This study proposed USLFA, an uncertainty‐suppressed long‐tailed learning method with lightweight feature adaptation for multi‐label CXR classification. In the primary CheXpert analysis, USLFA improved macro‐AUPRC, macro F1, and tail‐label AUPRC relative to the principal BCE and LDAM baselines. The incremental effect of uncertainty suppression was statistically supported for macro‐AUROC, macro‐AUPRC, and tail‐label AUPRC, but not for macro F1 relative to U Soft after Holm correction. Repeated patient‐level splits preserved the performance ordering, while MIMIC‐CXR‐JPG and the official CheXpert test set provided complementary evidence beyond the custom internal partition. The method remains a retrospective training approach, and broader multi‐institutional and prospective evaluation is required before clinical deployment.

## AUTHOR CONTRIBUTIONS STAEMENT

Lu Li conceived and designed the study, performed the experiments and statistical analyses, interpreted the results, and drafted and revised the manuscript. The author approved the final version of the manuscript.

## CONFLICT OF INTEREST STATEMENT

The author declares no competing interests.

## FUNDING INFORMATION

No funding was received for this study.

## Data Availability

The CheXpert and MIMIC‐CXR‐JPG datasets used in this study are available to qualified researchers through their official access procedures. The patient‐level split definitions, evaluation code, and processed prediction outputs generated in this study are available from the corresponding author upon reasonable request.
